# Fowl adenovirus infection and inclusion body hepatitis in Canada: genotyping trends from 2008 to 2024

**DOI:** 10.1177/10406387251412366

**Published:** 2026-01-21

**Authors:** Davor Ojkić, Jordyn Lopes, Christian Sandrock, Emily Rätsep, Emily Brouwer, Andrew Brooks, Tanya Rossi, Emily Martin

**Affiliations:** Animal Health Laboratory, University of Guelph, Guelph, Ontario, Canada; Animal Health Laboratory, University of Guelph, Guelph, Ontario, Canada; Animal Health Laboratory, University of Guelph, Guelph, Ontario, Canada; Animal Health Laboratory, University of Guelph, Guelph, Ontario, Canada; Animal Health Laboratory, University of Guelph, Guelph, Ontario, Canada; Animal Health Laboratory, University of Guelph, Guelph, Ontario, Canada; Animal Health Laboratory, University of Guelph, Guelph, Ontario, Canada; Animal Health Laboratory, University of Guelph, Guelph, Ontario, Canada

**Keywords:** fowl adenovirus, genotyping, hexon gene sequencing, inclusion body hepatitis, molecular characterization, surveillance

## Abstract

Between 2008 and 2024, fowl adenovirus (FAdV) genotypes were determined by hexon gene sequencing for 1,362 samples: 1,234 from 9 Canadian provinces and 128 samples from the United States. Most genotyped samples were from Ontario (681), followed by Alberta (243), Nova Scotia (116), British Columbia (77), Quebec (58), Saskatchewan (21), Manitoba (20), Newfoundland (16), and Prince Edward Island (2). Most samples (1,285) were related to inclusion body hepatitis (IBH); 77 samples were submitted for other reasons. Four FAdV genotypes (FAdV2, FAdV8a, FAdV8b, FAdV11) were associated with IBH-related submissions. Between 2008 and 2014, the most common strains associated with IBH outbreaks were FAdV11 and FAdV8a. However, since 2015, the identity of FAdVs involved in IBH outbreaks has shifted, with FAdV8b becoming the most frequent IBH-associated strain, largely displacing FAdV8a and FAdV11. In a much smaller group of 77 samples from non-IBH submissions, 10 FAdV genotypes were detected: FAdV1, FAdV2, FAdV3, FAdV4, FAdV6, FAdV7, FAdV8a, FAdV8b, FAdV9, and FAdV11. Although FAdV4 is a recognized causative agent of hepatitis–hydropericardium syndrome worldwide, no association with clinical disease was reported in the birds included in our study. Our comprehensive 17-y analysis of FAdV circulation patterns will support the development of control measures and vaccination programs to reduce the impact of FAdV-related outbreaks.

Fowl adenoviruses (**FAdVs**; family *Adenoviridae*, genus *Aviadenovirus*) that affect chickens include 12 serotypes (FAdV1–7, 8a, 8b, and 9–11).^11^ Infections with many FAdVs are subclinical and not associated with overt disease presentations. However, certain FAdV serotypes have the potential to cause significant health issues in chickens.^15^ In broiler flocks affected by inclusion body hepatitis (**IBH**), FAdV2, FAdV8a, FAdV8b, and FAdV11 are detected most frequently.^29^ FAdV4 infection—related to hepatitis-hydropericardium syndrome (**HHS**)—is widespread throughout Asia, Africa, South America, and Mexico, but appears less frequently in Europe.^6^ In the United States, FAdV4 has been identified in both commercial and non-commercial flocks with hepatitis and sporadic hydropericardium.^31^ In contrast, in Canada, FAdV4 has been detected on multiple occasions in cloacal swabs from subclinical broilers and broiler breeders, as well as in non-commercial chicken flocks with ambiguous clinical signs without hepatic involvement.^3,7,21^ Sporadic gizzard erosion outbreaks, first described in Japan, have been associated most frequently with FAdV1, and occasionally with FAdV4, FAdV8a, and FAdV8b infection.^32^ FAdV-associated ventriculitis with intranuclear inclusions has been reported in broilers and layers in multiple countries, including Poland, Italy, Germany, Korea, the United Kingdom, the United States, Morocco, and Canada.^13,25,32^

IBH can be a devastating disease in broiler chickens, often causing high mortality and significant economic losses for poultry producers. IBH was first described in Connecticut in 1963 as a hepatitis with distinctive intranuclear inclusion bodies in hepatocytes. However, an etiologic agent related to pathology findings could not be isolated in embryonated chicken eggs at that time.^10^ Only a decade later, agents present in the livers of affected chickens provided conclusive evidence—based on analyses of their physical, chemical, and biological properties—that adenovirus was the cause of IBH.^26^ In Canada, IBH submissions from flocks with high mortality increased markedly from 2000 to 2006; ~40% of those were caused by FAdV11, 40% by FAdV8a, and the remaining 20% involved FAdV8b and FAdV2.^21^ We describe FAdV detections at the Animal Health Laboratory (**AHL**; University of Guelph, Guelph, Ontario, Canada) between 2008 and 2024 in samples from 9 Canadian provinces and from the United States.

## Materials and methods

### Samples

From 2008 Jan 1 to 2024 Dec 31, we tested 5,719 samples by FAdV PCR at the AHL. Of these, 1,810 samples were from research projects and species other than chickens and were excluded from our study. From the remaining 3,909 samples submitted for diagnostic and surveillance purposes, 1,362 FAdV-positive samples were genotyped upon submitters’ requests. These included 1,234 samples from 9 Canadian provinces and 128 from the United States (**Table 1**). The dataset comprised 1,288 samples from broilers, 45 samples from broiler breeders, 28 samples from non-commercial flocks, and 1 sample from a layer flock.

**Table 1.** Geographic origin of samples used for fowl adenovirus genotyping.

Country/Province	IBH	Non-IBH	Total
Canada			
Alberta	238	5	243
British Columbia	77	0	77
Manitoba	20	0	20
Newfoundland	16	0	16
Nova Scotia	116	0	116
Ontario	612	69	681
Prince Edward Island	2	0	2
Quebec	57	1	58
Saskatchewan	21	0	21
United States	126	2	128
Total	1,285	77	1,362

IBH = inclusion body hepatitis.

### Criteria

We classified 1,285 genotyped samples as IBH-related, given that they were PCR-positive liver-derived samples (e.g., liver tissue, liver swabs, virus isolates) accompanied by IBH-compatible clinical history and/or gross or histologic lesions. We classified 77 samples as non-IBH because, although FAdV PCR-positive, they lacked histologic lesions or clinical history consistent with IBH, and were derived from non-hepatic tissues (e.g., cloacal swabs, lung, ventriculus, meconium). One sample per submission was genotyped.

### Nucleic acid extraction and PCR

We used 2 commercial kits for nucleic acid extraction (MagMAX-96 viral RNA isolation kit, with a MagMAX Express-96 processor, ThermoFisher; MagNA Pure 96 DNA and Viral NA small volume kit, with a MagNA Pure 96 system, Roche). From 2008 to 2012 Aug, we utilized a conventional PCR to detect all FAdVs.^16^ From 2012 Aug to 2024 we used a triplex real-time PCR (**Table 2**) to differentiate between FAdV2/3/9/11 (FAdV PCR D) and 6/7/8a/8b (FAdV PCR E). An armored enterovirus internal control (Asuragen) was added to the nucleic acid kit lysis buffer. PCR amplification and detection (Light Cycler 480; Roche) used the parameters in **Table 3**. Samples with a Ct <37 were considered positive, and samples with Ct ≥37 were considered inconclusive. If no amplification was detected (e.g., no Ct value was produced), the sample was considered negative.

**Table 2.** Triplex real-time PCR primer and probe sequences for fowl adenovirus PCR assays.

Oligo name	Target	Sequence 5′–3′	Final conc., µM
FAdVE_F	Hexon gene: FAdV6/7/8a/8b	GGGTGATGAAAGCBAACAGA	0.5
FAdVE_R		TCGTGGTAYAGGAGGTTGATGA	0.5
FAdVE_Pr		FAM-CCAAYTACATCGGGTTCCGTGACAA	0.25
FAdVD_F	Hexon gene: FAdV2/3/9/11	GTCATGGGAGTCGAAGACTTTAG	0.5
FAdVD_R		CCTTCATGACGCCGGTATT	0.5
FAdVD_Pr		HEX-CCGCCGACCGAATACTCAGAAGTG	0.25
FAdV_52k_F2	52 K gene: all FAdVs	ATGGCKCAGATGGCYAAGGC	0.5
FAdV_52k_R		AGCGCCTGGGTCAAACC	0.5
FAdV_52k_Pr		TexRd-CAGATGWCTGACGCSGASTACATGTT	0.25
Entero_31_For	Enterovirus 5′-UTR: internal control	ATGCGGCTAATCCCAACCT	0.2
Entero_31_Rev		CGTTACGACAGGCCAATCACT	0.2
Entero_LNA_Pr		Cy5-CA+G+GTGGTCA+C+AAAC	0.06

conc. = concentration.

**Table 3.** Cycling parameters for fowl adenovirus PCR assays.

Step	Temperature, °C	Hold (mm:ss)	Ramp, °C/s	Cycles (×)
Reverse transcription	45	10:00	4.4	1
Activation	95	10:00	4.4	1
Denaturation	94	0:05	2.2	45
Annealing/extension	60	1:00	2.2	45
Cool	40	10:00	2.2	1

### Genotyping

We conducted PCR amplification of the hexon protein L1 loop (One step RT-PCR kit, Qiagen; Biometra T3 thermocycler, Analytik Jena) with primers described previously.^17^ Nucleotide (nt) sequences of amplicons were determined at the Laboratory Services Division (University of Guelph, Guelph, Ontario, Canada). For sequence assembly, we utilized the SeqMan Pro module of Lasergene software (v.17.5.0; DNAStar) and Geneious Prime software (v.2025.1.2; GraphPad) to calculate nt sequence identities.

## Results

The number of samples we tested by FAdV PCR varied, from only 16 samples in 2008 to 1,237 samples in 2023. Of 3,909 samples, 2,741 (70.1%) were positive. Among the 295 samples tested using conventional PCR, 215 (72.3%) were positive, while 2,526 (69.9%) of the 3,614 samples tested using triplex PCR were positive. Signals positive only for FAdV PCR E were found in 1,196 (33.1%) samples, while 249 samples (6.9%) were positive only for FAdV PCR D, and 96 (2.7%) were positive only on the cross-reactive FAdV 52k PCR. Mixed signals were detected in 985 (27.3%) of the samples tested.

**Figure 1.**
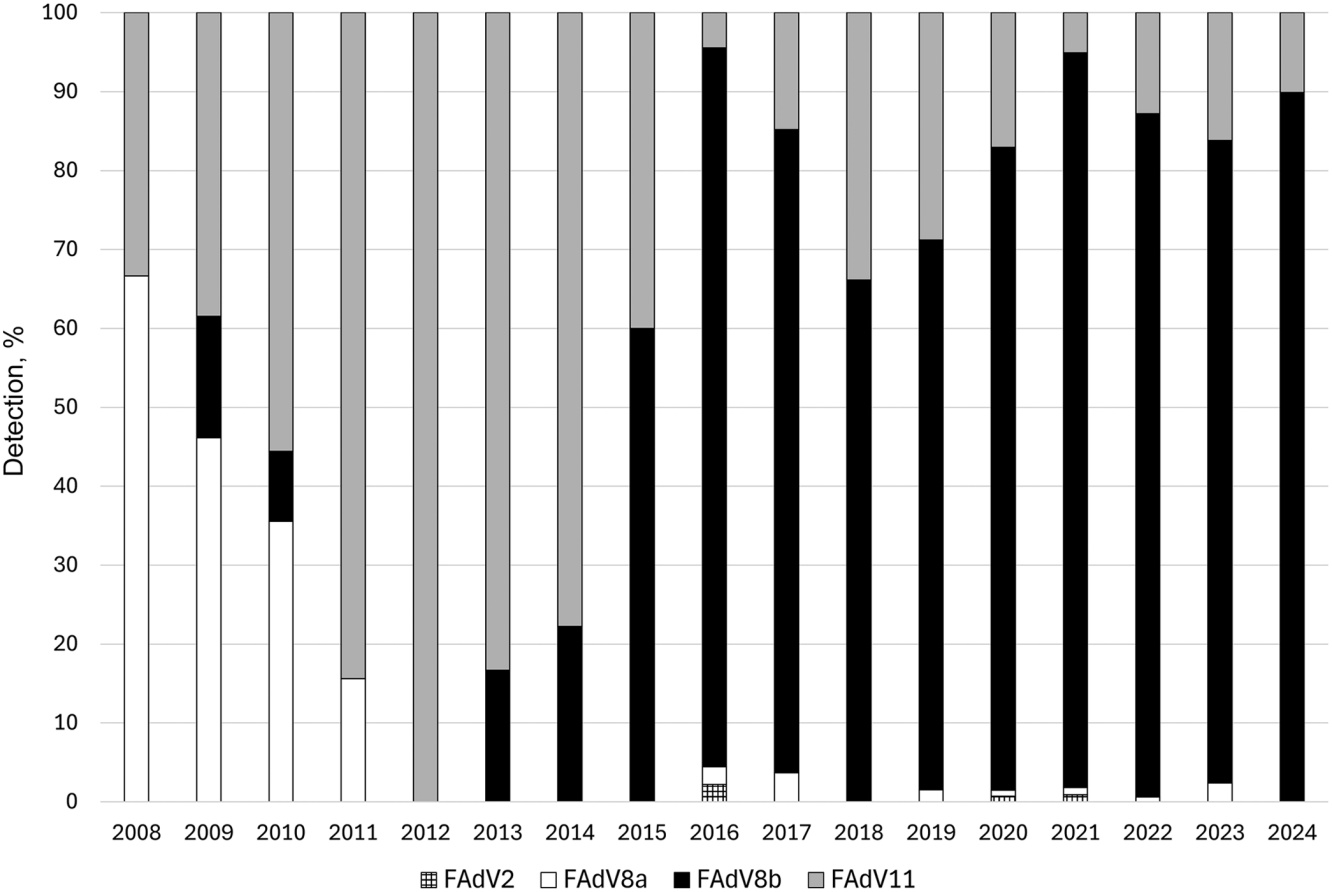
Detection of inclusion body hepatitis-related fowl adenovirus (FAdV) genotype, 2008–2024.

We genotyped 1,362 samples and submitted the nt generated sequences to GenBank (PV880996–PV882357). Of the samples, 1,285 (94.3%) were from flocks affected by IBH; 77 (5.7%) were from flocks without IBH. Between 2008 and 2024, infection with 4 genotypes of FAdVs was associated with IBH: 992 (77.2%) samples were FAdV8b, 241 (18.8%) were FAdV11, 48 (3.7%) were FAdV8a, and 4 (0.3%) were FAdV2 (**Fig. 1**). Before 2014, the predominant FAdV8b strain was related to an isolate from 2004 (AHL04-53357-74, GenBank EF685508). Since 2014, coinciding with an increased frequency of field reports indicating high mortality, the AHL04-53357-74 strain has been almost completely displaced by 2 strains: AHL16-049095 (GenBank PV881385) and AHL18-057921 (GenBank PV881459). These 2 strains were 97.7% identical at the nt level but were clearly distinguishable as separate viral populations. In 2024, these 2 FAdV8b strains were detected in 240 of 267 (89.9%) genotyped samples. Of 130 IBH samples from 2008–2014, we most frequently detected FAdV11 in 84 (64.6%) samples. From 2015 to 2024, we most frequently detected FAdV8b in 983 (85.1%) of 1,155 IBH-related samples (**Table 4**).

**Table 4.** Fowl adenovirus genotypes and strains detected in samples related to inclusion body hepatitis.

Genotype	Strain	2008	2009	2010	2011	2012	2013	2014	2015	2016	2017	2018	2019	2020	2021	2022	2023	2024	Total
FAdV2	US_685									1				1	1				3
FAdV2	US_P7A														1				1
FAdV8a	US_T8-A	10	6	16	5						1		1		2	1	2		44
FAdV8a	US_TR59									1				1			2		4
FAdV8b	ON_AHL16-049095							2	2	11	16	38	26	43	85	90	57	100	470
FAdV8b	ON_AHL18-057921									27	4	3	19	67	114	51	79	140	504
FAdV8b	SK_AHL04-53357-122									1									1
FAdV8b	SK_AHL04-53357-74		2	4			1		1	2	2		1		3	1			17
FAdV11	US_1047	5	5	25	27	10	5	7	2	2	4	21	19	23	11	21	27	27	241
Total		15	13	45	32	10	6	9	5	45	27	62	66	135	217	164	167	267	1,285

Blank cells indicate a detection frequency of 0.

On the other hand, in 77 non-IBH samples, we detected 10 FAdV genotypes. Most samples, 23 (29.9%), were FAdV1; 13 (16.9%) were FAdV8a; 12 (15.6%) were FAdV4; 10 (13.0%) were FAdV11; 6 (7.8%) were FAdV3; 6 (7.8%) were FAdV8b; 3 (3.9%) were FAdV9; 2 (2.6%) were FAdV7; and 1 (1.3%) each of FAdV2 and FAdV6 (**Table 5**). Of the 12 FAdV4 detections, 6 (50.0%) were from broiler breeders, and 6 (50.0%) were from non-commercial flocks. Of the 2 samples from chickens with gizzard erosions that tested positive for FAdV, 1 was from broiler breeders with FAdV8a and 1 was from commercial layers with FAdV1.

**Table 5.** Fowl adenovirus genotypes and strains detected in samples not related to inclusion body hepatitis.

Genotype	Strain	2009	2010	2015	2016	2017	2021	2022	2023	2024	Total
FAdV1	US_CELO	16			1	1			2	3	23
FAdV2	US_685					1					1
FAdV3	US_SR49	1			4	1					6
FAdV4	US_J2A	6		2	2	2					12
FAdV6	US_CR119				1						1
FAdV7	US_Y36	1			1						2
FAdV8a	US_T8-A		1		1						2
FAdV8a	US_TR59	6			2	3					11
FAdV8b	AU_Esurient	2									2
FAdV8b	ON_AHL16-049095				1	1					2
FAdV8b	ON_AHL18-057921									1	1
FAdV8b	SK_AHL04-53357-74		1								1
FAdV9	US_A-2A				1	2					3
FAdV11	US_1047	1			1		1	3		2	8
FAdV11	US_380				1	1					2
Total		33	2	2	16	12	1	3	2	6	77

Blank cells indicate a detection frequency of 0.

## Discussion

An increase in incidence of IBH outbreaks caused by FAdV infection was reported by the AHL across 9 provinces in Canada between 2004 and 2006.^21^ In 2010, widespread vaccination of broiler breeders was introduced in Ontario with bivalent, autogenous vaccines containing FAdV8a and FAdV11. In the years following the introduction of vaccination, the incidence of IBH outbreaks declined. However, since 2015, an increase in IBH outbreaks has been observed in Canada and worldwide.^22,29^ We genotyped FAdVs in Canada from samples that were submitted to the AHL from 2008 to 2024 and noted a shift in genotype representation. Between 2008 and 2014, FAdV11 and FAdV8a were the most common strains associated with IBH outbreaks. However, in 2014, a new FAdV8b strain was first detected in 2 samples from Nova Scotia, Canada. Since 2015, FAdV8b has largely displaced FAdV8a and FAdV11 in IBH-related submissions. Of 128 US-origin samples that we received from 2016 to 2021, most, 117 (84.7%), were FAdV8b. Reports describing the introduction of new FAdV8b strains and/or the involvement of FAdV8b as a main IBH-associated strain have been emerging globally—from multiple continents and countries, including China, Iran, Spain, Brazil, Japan, Hungary, Türkiye, South Korea, Malaysia, Egypt, Canada, Poland, and Morocco.^1,2,4,5,12,14,18–20,22–24,27,28^

In 28 samples from non-commercial flocks, 10 FAdV genotypes were detected, but none were from birds with IBH and were not considered primary disease-causing agents. Of 49 non-IBH samples from commercial flocks, only 2 were clearly associated with clinical disease or gizzard erosions. Detection of FAdV4 has been reported in both commercial and non-commercial flocks in Canada and the United States.^3,7,22,31^ However, FAdV4 infection has not been associated with clinical presentation of HHS in broilers in Canada. An experimental inoculation with a high dose of FAdV4 (2 × 10^8^ pfu/chick) did not result in disease in 10-d-old specific-pathogen-free Leghorn chicks.^8^ We detected FAdV4 infection in subclinical broiler breeders and in non-commercial flocks with no signs of HHS or IBH. This suggests that the FAdV4 strains circulating in Canada are either non-hepatopathogenic or that other (yet unrecognized) co-factors necessary for disease induction are absent.

To date, there are no commercial, licensed live vaccines against IBH in North America, highlighting the need for alternative and adaptive strategies to control and reduce the severity of IBH outbreaks in broilers.^9^ Although autogenous inactivated vaccines have been used widely in broiler breeders throughout Canada, they sometimes provide inconsistent levels of passive immunity. Increased biosecurity measures that limit the natural exposure of broiler breeders may inadvertently reduce the generation and transmission of passive maternal antibodies, thereby increasing the risk to their offspring.^30^

Our findings are subject to limitations from sample collection bias, given that collection was passive and most samples came from Ontario poultry producers—reflecting both the size of the poultry industry in Ontario and the location of our laboratory. Nevertheless, we anticipate that the samples generally reflected the presumed impact of IBH-related field issues in Canada. The IBH situation in Canada may also be compared, to some extent, with that in the United States. From 2016 to 2021, we received 128 samples from the United States, However, no samples were received between 2022 and 2024, reportedly because of the introduction of genotyping services by local US laboratories. Given the size of the poultry industry in Canada and the geographic distribution of sample origins, we believe that our study offers robust data on FAdV circulation patterns across Canada. Our findings may inform effective strategies to minimize and control FAdV-associated outbreaks.
